# Association Between Periodontal Disease and Blood Biomarkers in U.S. Adults: A Cross-Sectional Study

**DOI:** 10.3390/biomedicines13122991

**Published:** 2025-12-05

**Authors:** Duaa Alshammari, Yousef Alharbi, Aseel Alshuaib, Zainab Haidar, Fahad AlAli, Yousef Alenezi, Hend Alqaderi, Basel Hamoud

**Affiliations:** 1 Kuwait Ministry of Health, Kuwait City 15462, Kuwait; 2 Department of Public Health, School of Dental Medicine, Tufts University, Boston, MA 02111, USA; hend.alqaderi@tufts.edu; 3 Dasman Diabetes Institute, Kuwait City 15462, Kuwait

**Keywords:** periodontal disease, blood biomarkers, WBC, serum albumin, MCHC

## Abstract

**Background:** Periodontal disease (PD) is a chronic inflammatory condition linked to systemic immunologic and metabolic alterations. This study evaluated associations between PD and three routinely measured blood biomarkers—white blood cell (WBC) count, serum albumin, and mean corpuscular hemoglobin concentration (MCHC)—using data from 4669 adults aged ≥30 years in the 2013–2014 National Health and Nutrition Examination Survey (NHANES). **Methods:** PD was defined dichotomously according to the Centers for Disease Control and Prevention/American Academy of Periodontology (CDC/AAP) surveillance criteria. All analyses incorporated NHANES sampling weights, strata, and primary sampling units. Weighted descriptive statistics compared characteristics by PD status. Stepwise survey-weighted logistic regression examined associations between biomarkers and PD, adjusting for sociodemographic, behavioral, and health-related confounders. Restricted cubic splines assessed nonlinearity, and biomarker effects were additionally scaled per standard deviation (SD). **Results:** Higher WBC counts (OR = 1.08; 95% CI: 1.04–1.11) and higher MCHC values (OR = 1.14; 95% CI: 1.06–1.22) were positively associated with PD, whereas serum albumin showed an inverse association (OR = 0.76; 95% CI: 0.62–0.93). Spline models demonstrated significant nonlinear components for all biomarkers, and SD-scaled estimates confirmed consistent gradients. **Conclusions:** These findings support links between periodontal inflammation and systemic hematologic alterations. Longitudinal studies are needed to clarify underlying mechanisms.

## 1. Introduction

Periodontal disease (PD) is a prevalent chronic inflammatory condition that affects the supporting structures of the teeth, including the gingiva, periodontal ligament, and alveolar bone [1,2,3]. It is one of the most common oral diseases globally and is a leading cause of tooth loss in adults [1,2,3]. According to the Centers for Disease Control and Prevention (CDC), about 42% of U.S. adults aged 30 years and older exhibit signs of periodontal disease, with prevalence increasing with age [4]. Research has shown that PD has been a potential contributor to systemic inflammation and has been linked to various chronic conditions, including diabetes, cardiovascular disease, and chronic kidney disease [3]. Systematic reviews and meta-analyses further demonstrate that individuals with rheumatoid arthritis exhibit a higher prevalence of periodontitis, and periodontal therapy can reduce systemic inflammatory biomarkers, highlighting an important oral–systemic inflammatory axis [5,6,7,8].

The pathogenesis of PD is initiated by sub-gingival microbial biofilms that trigger a host immune response, resulting in tissue destruction when left unresolved [9]. This immune response is not only confined to the oral cavity but may also elicit systemic alterations in inflammatory and metabolic markers detectable in peripheral blood [10,11]. As such, blood-based biomarkers have gained attention as non-invasive indicators of the systemic impact of PD [10,11].

Among these, white blood cell (WBC) count is a widely used measure of systemic inflammation and immune activation [12,13]. Elevated WBC counts have been linked to periodontal tissue destruction, reflecting the mobilization of leukocytes in response to chronic microbial challenge [14,15]. Serum albumin, the most abundant plasma protein, serves as a negative acute-phase reactant, and its levels may reflect both nutritional depletion and the severity of systemic inflammation [16,17]. Studies have shown that serum albumin levels may decrease during systemic inflammation due to cytokine-mediated suppression of hepatic synthesis [18]. Additionally, lower serum albumin concentrations have been reported in individuals with chronic inflammatory diseases, including periodontitis [19]. 

Mean corpuscular hemoglobin concentration (MCHC), which represents the average hemoglobin concentration within red blood cells, provides insight into erythrocyte hemoglobinization and iron metabolism [20,21]. Elevated MCHC may reflect subtle shifts in iron handling and redox balance, as hemoglobin concentration within red cells is influenced by systemic iron metabolism and inflammatory signals [20,21]. Alterations in MCHC are observed in anemia of chronic disease, a condition influenced by sustained inflammatory cytokine activity, and have been associated with periodontitis in clinical studies [22].

Despite multiple studies linking periodontal status to systemic inflammatory markers, these investigations typically assess isolated indices (e.g., leukocyte counts or CRP) in convenience or disease-specific cohorts. To date, no NHANES-based study has jointly examined WBC, serum albumin, and MCHC in relation to periodontal disease [23,24]. In addition, although several reviews and studies have explored the pathophysiology of anemia of chronic disease, disturbances in iron metabolism, and the relationship between anemia and periodontitis, most have focused on broader hematologic parameters such as hemoglobin, hematocrit, and red blood cell count, without specifically addressing MCHC [25,26].

To address this gap, the present study aims to investigate the association between periodontal disease and three systemic blood biomarkers, including WBC count, serum albumin, and MCHC, using data from the 2013–2014 National Health and Nutrition Examination Survey (NHANES). This analysis looks to better understand the systemic inflammatory and metabolic profile of individuals with periodontal disease, and to explore whether these biomarkers may serve as potential indicators of oral–systemic health interaction in the general U.S. adult population. We hypothesize that individuals with periodontal disease exhibit higher WBC counts, lower serum albumin levels, and higher MCHC values, potentially due to systemic inflammatory and hematologic responses associated with chronic periodontal inflammation.

## 2. Materials and Methods

### 2.1. Study Population

In this cross-sectional study, data was utilized from the 2013–2014 NHANES, a nationally representative survey of the non-institutionalized civilian population in the United States. The NHANES, conducted by the National Center for Health Statistics, Centers for Disease Control and Prevention, employs a complex probability sampling design involving multiple stages, stratification, and clustering. The NHANES sampling strategy includes stratification by demographic and geographic characteristics, cluster sampling within primary sampling units (PSUs), oversampling of specific population subgroups (e.g., non-Hispanic Black, Hispanic, Asian, and older adults) to ensure statistical reliability for these groups, and use of sampling weights in analyses to adjust for unequal probabilities of selection, nonresponse, and post-stratification.

The NHANES collects data on various health outcomes and explanatory variables through a combination of interviews, laboratory tests, and clinical examinations. As a component of the NHANES, trained and calibrated dental professionals conducted comprehensive periodontal examinations on survey participants aged 30 years and older within the Mobile Examination Centers. All participants provided written informed consent. This study used publicly available, de-identified NHANES data and was therefore classified as secondary data analysis, exempt from Institutional Review Board (IRB) review. The study followed the STROBE (Strengthening the Reporting of Observational Studies in Epidemiology) guidelines. Nationally representative estimates were obtained by incorporating NHANES sample weights, strata, and primary sampling units (PSUs) in all analyses.

#### Eligibility Criteria

**Figure 1:**
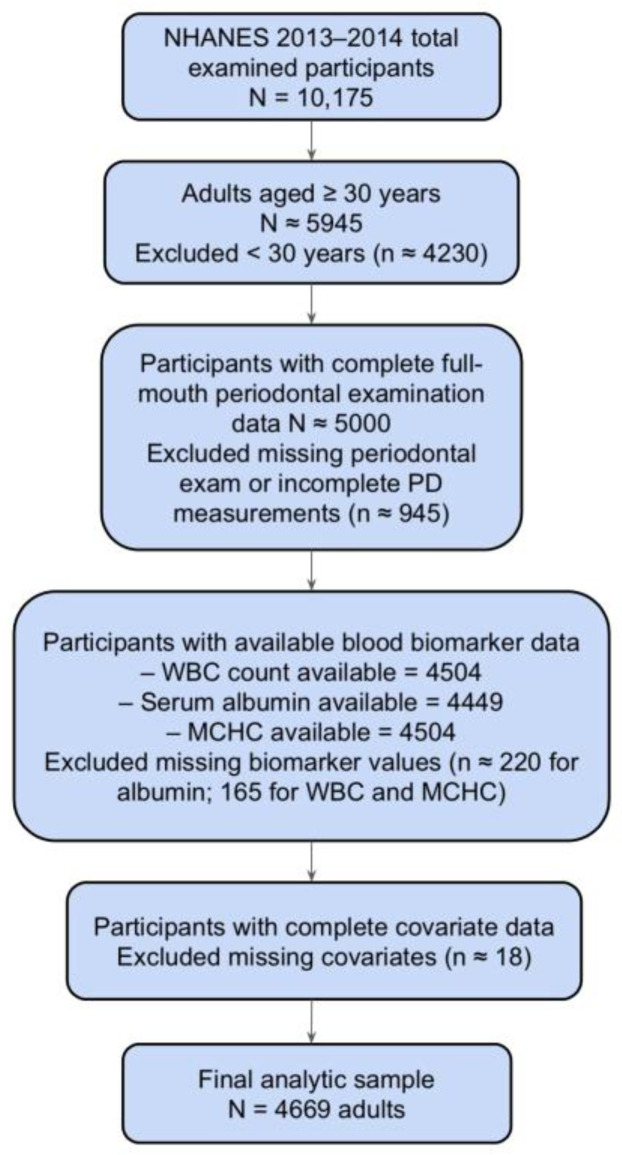
The flowchart of participant inclusion and exclusion criteria follows STROBE guidelines.

From the 2013–2014 NHANES cycle, adults aged ≥30 years who completed a full-mouth periodontal examination and had available data on the biomarkers of interest (WBC count, serum albumin, and MCHC) and key covariates were eligible for inclusion. Participants were excluded if they lacked periodontal examination data, had missing values for any of the three biomarkers, were missing key covariates (age, sex, race/ethnicity, education, smoking status, comorbidity status, or income-to-poverty ratio), or were pregnant. The final analytic sample consisted of 4669 participants. The selection process is summarized in Figure 1.

### 2.2. Periodontal Examination

The NHANES study of 2013 to 2014 was conducted using a full-mouth periodontal examination (FMPE) among individuals aged ≥30 years who did not have a health condition that required antibiotic prophylaxis before periodontal testing. The FMPE was conducted by calibrated dental examiners with the intent to produce gold-standard assessments for clinical attachment loss (AL). For this reason, direct measurements of both the distance between the cemento-enamel junction and the free gingival margin (CEJ-FGM) and the probing depth (PD) were made at each site. All measurements were taken at six sites (mesiobuccal, midbuccal, distobuccal, mesiolingual, midlingual, and distolingual) of all teeth, with the exclusion of third molars. All calculations were rounded to the lower whole millimeter, and clinical AL was calculated based on the recorded measurements.

### 2.3. Definition of the Dependent Variable: Periodontal Disease

Periodontal disease was defined as a binary variable (periodontitis vs. no periodontitis) based on clinical attachment loss (CAL) and probing pocket depth (PPD) measurements obtained during the full-mouth periodontal examination. Periodontitis was classified according to the Centers for Disease Control and Prevention/American Academy of Periodontology (CDC/AAP) case definition for population surveillance, in which individuals are considered to have periodontitis if they present with ≥1 site with CAL ≥ 3 mm and PPD ≥ 4 mm, or ≥2 interproximal sites with CAL ≥ 4 mm [27]. For analytic purposes, we dichotomized periodontal status into “periodontitis” versus “no periodontitis.” Participants were classified as having periodontitis if they met CDC/AAP criteria for any severity category (mild, moderate, or severe). Those who did not meet any CDC/AAP threshold were classified as having no periodontitis. This binary operationalization is consistent with prior NHANES-based epidemiologic studies [28].

### 2.4. Description of Independent Variable: Blood Biomarkers

#### 2.4.1. White Blood Cell Count

White blood cell (WBC) count was obtained from the NHANES Complete Blood Count (CBC) laboratory component (variable IBXWBCSI). WBC was treated as a continuous variable, reported in 10^3^ cells/µL of whole blood. Measurements were performed using an automated hematology analyzer (Coulter^®^ DxH 800, Beckman Coulter, Inc., Brea, CA, USA). 

#### 2.4.2. Serum Albumin

Serum albumin was obtained from the NHANES Biochemistry Profile laboratory component (variable IBXSAL). It was treated as a continuous variable, reported in grams per deciliter (g/dL). Measurements were performed using a colorimetric bromocresol purple dye-binding method on a Roche/Hitachi Modular P Chemistry Analyzer (Roche Diagnostics, Indianapolis, IN, USA).

#### 2.4.3. Mean Corpuscular Hemoglobin Concentration

Mean corpuscular hemoglobin concentration (MCHC) was obtained from the NHANES Complete Blood Count (CBC) laboratory component (variable IBXMC). It was treated as a continuous variable, reported in grams per deciliter (g/dL). MCHC was calculated by the Coulter^®^ DxH 800 Hematology Analyzer as part of the automated complete blood count (Beckman Coulter Inc., Brea, CA, USA). 

### 2.5. Potential Confounding Variables

In this study, several demographic and health-related variables were categorized for analysis. Age was grouped into four categories: 30–34 years, 35–49 years, 45–64 years, and 65 years or older. Sex was classified as a binary variable (male or female) while race was treated as a nominal variable with categories including “Non-Hispanic White”, “Non-Hispanic Black”, “Hispanic”, “Non-Hispanic Asian”, and “Other” races. Education level was divided into three categories: less than high school, high school graduate or equivalent (including GED), and more than high school education. The variable “Any disease” was created as a composite indicator of major chronic conditions, coded as “1” if participants reported at least one physician-diagnosed systemic disease and “0” otherwise. Included conditions were cardiovascular disease (congestive heart failure, coronary heart disease, heart attack, or stroke), diabetes, chronic kidney disease, liver disease, cancer, chronic bronchitis, or emphysema, based on the NHANES Medical Conditions (MCQ), Diabetes (DIQ), and Kidney (KIQ) questionnaires [29].

### 2.6. Statistical Methods

Descriptive statistics were used to summarize participant characteristics. Survey-weighted proportions were compared using Rao–Scott adjusted χ^2^ tests for categorical variables, and survey-weighted means were compared using svy: mean for continuous variables. All analyses incorporated the NHANES complex, multistage probability sampling design and used MEC examination weights (WTMEC2YR), accounting for strata (SDMVSTRA) and primary sampling units (SDMVPSU) to ensure nationally representative estimates.

A stepwise multivariable logistic regression procedure was used to identify routine blood biomarkers associated with periodontal disease while maintaining model parsimony. Demographic and behavioral covariates (age, sex, race/ethnicity, education, family income-to-poverty ratio, smoking), as well as comorbidity status (“any disease”), were retained in all models based on established associations with periodontal disease. Candidate biomarkers entered the model at *p* < 0.20 and were removed if ≥0.20.

Multicollinearity was evaluated using survey-weighted Pearson correlation matrices, and variables with strong correlations (r > 0.5) were reviewed for redundancy based on clinical interpretability. Where necessary, correlated laboratory measures were removed.

Survey-weighted design corrections were applied to all inferential analyses, including descriptive comparisons and multivariable regression modeling, to ensure valid variance estimation and correct standard errors.

Missing data were addressed using complete-case analysis. The number of non-missing observations and percentage of missing values for each analytic variable are summarized in Supplementary Table S1. Missingness was <5% for all blood biomarkers and <1% for sociodemographic and behavioral covariates. Given the very low degree of missingness and the complex survey structure of NHANES, multiple imputations were not performed.

Smoking was modeled as a binary confounder (Yes/No) rather than as a categorical variable (never/former/current). This decision was based on two considerations. First, smoking was not an exposure of interest, and the objective of the study was not to quantify the dose–response relationship between smoking and periodontitis, which has already been extensively documented in the literature. Second, detailed smoking history (e.g., pack-years) exhibits substantial missingness in NHANES 2013–2014, and reclassification into multilevel categories would introduce additional missing data and reduce analytic stability. To ensure that this coding decision did not mask effect modification, we tested interaction terms between smoking and each biomarker (WBC × smoking, albumin × smoking, and MCHC × smoking). None of the interaction terms were statistically positive, indicating that smoking did not modify the biomarker–periodontitis associations.

Linearity of continuous biomarkers (WBC count, serum albumin, and MCHC) with respect to periodontal disease was assessed using restricted cubic splines constructed with knots at the 10th, 50th, and 90th percentiles of each biomarker. Spline-expanded terms were fit in survey-weighted logistic regression models, and the joint significance of spline components was evaluated using Wald tests. Evidence of nonlinearity was detected for all three biomarkers; therefore, spline terms were retained in sensitivity analyses. For the primary adjusted models and to enhance interpretability, biomarker effects were additionally expressed per one standard deviation (SD). Because odds ratios are exponentiated effects, SD-scaled biomarkers may yield numerically large ORs even when the underlying linear effect size is modest. This reflects the mathematical behavior of logistic models when predictors are rescaled and does not imply implausibly large biological effects.

Associations were expressed as odds ratios (ORs) with 95% confidence intervals (CIs). A two-sided *p* < 0.05 was considered statistically significant. All analyses were conducted using STATA 17.0 (StataCorp LLC, College Station, TX, USA).

### 2.7. Directed Acyclic Graph (DAG)

A directed acyclic graph (DAG) was constructed to illustrate the assumed causal relationships between periodontal disease, systemic blood biomarkers, and potential confounders. Age, sex, race/ethnicity, education, family income-to-poverty ratio, body mass index (BMI), smoking status, and comorbidity burden (“any disease”) were modeled as potential common causes of both periodontal disease and the blood biomarkers. These variables were therefore included in the minimally sufficient adjustment set to control for confounding. Systemic inflammation and oxidative stress, represented conceptually by markers such as C-reactive protein (CRP), were considered mediating mechanisms linking periodontal disease to hematologic alterations (particularly WBC and serum albumin). Consequently, CRP and related inflammatory biomarkers were not adjusted for in the primary models to prevent overadjustment. The DAG is provided in Supplementary Figure S1.

## 3. Results

Table 1 summarizes the survey-weighted demographic, socioeconomic, and behavioral characteristics of U.S. adults aged ≥30 years by periodontal disease status. Of the 4669 adults included in the analytic sample, 2116 (45.3%) had periodontitis. Periodontitis was more prevalent among males, adults aged 45–64 years, Non-Hispanic Black and Hispanic individuals, those with lower educational attainment, lower income-to-poverty ratios, and current smokers (all *p* < 0.05). The distribution of overall comorbidity burden (“any disease”) did not differ significantly between groups (*p* = 0.149).

Table 2 presents weighted biomarker levels. Individuals with periodontal disease had significantly higher WBC counts (*p* = 0.002) and lower serum albumin levels (*p* = 0.002) compared with those without periodontal disease, while MCHC demonstrated a borderline, non-significant elevation among participants with periodontal disease (*p* = 0.052). These survey-weighted descriptive findings provided the basis for covariate selection and informed subsequent multivariable modeling.

Table 3 presents the adjusted stepwise survey-weighted logistic regression model examining the associations between blood biomarkers, demographic factors, and periodontal disease. Higher white blood cell (WBC) counts were positively associated with periodontal disease (OR = 1.08, 95% CI: 1.04–1.11, *p* < 0.001), indicating greater periodontal disease burden among individuals with elevated systemic leukocyte levels. Mean corpuscular hemoglobin concentration (MCHC) was also positively associated with periodontal disease (OR = 1.14, 95% CI: 1.06–1.22, *p* < 0.001). In contrast, serum albumin exhibited an inverse association (OR = 0.76, 95% CI: 0.62–0.93, *p* = 0.011), with lower albumin levels linked to greater odds of periodontal disease, consistent with its role as a negative acute-phase reactant. Female participants had lower odds of periodontal disease compared with males (OR = 0.55, 95% CI: 0.48–0.63, *p* < 0.001). Odds increased markedly with age, particularly among individuals aged 45–64 years (OR = 1.95, 95% CI: 1.53–2.49, *p* < 0.001) and ≥65 years (OR = 1.74, 95% CI: 1.35–2.26, *p* < 0.001), compared with those aged 30–34 years. Socioeconomic gradients were evident: participants with higher income (≥400% FPL) had substantially lower odds of periodontal disease (OR = 0.61, 95% CI: 0.51–0.72, *p* < 0.001) relative to those below 138% FPL. Education also demonstrated a protective effect, with individuals who completed some college or higher education showing lower odds of disease (OR = 0.73, 95% CI: 0.62–0.87, *p* = 0.001). Marked racial/ethnic disparities were noted. Compared with non-Hispanic Whites, the odds of periodontal disease were significantly higher among non-Hispanic Black (OR = 1.93, 95% CI: 1.60–2.32, *p* < 0.001), Hispanic (OR = 1.69, 95% CI: 1.42–2.01, *p* < 0.001), and non-Hispanic Asian adults (OR = 1.81, 95% CI: 1.46–2.25, *p* < 0.001). Smoking was positively associated with periodontal disease (OR = 1.31, 95% CI: 1.15–1.50, *p* < 0.01), consistent with its established role as a behavioral risk factor. Finally, the composite comorbidity indicator (“any disease”) was not significantly associated with periodontal disease (OR = 0.91, 95% CI: 0.78–1.05, *p* = 0.19).

Restricted cubic spline regression identified statistically positive nonlinear associations for all three biomarkers in survey-weighted models. Specifically, WBC count demonstrated a positive nonlinear component (nonlinear *p* = 0.010), serum albumin showed evidence of nonlinearity (*p* = 0.028), and MCHC also exhibited a nonlinear pattern (*p* = 0.031), confirming deviations from linearity in their relationships with periodontal disease.

In supplementary analyses, we evaluated the association between periodontal disease and each biomarker scaled per one standard deviation (SD) to enhance interpretability. Table S2 reports these SD-standardized estimates. A one-SD increase in white blood cell count (SD = 2.29 × 10^3^ cells/µL) was associated with markedly higher odds of periodontal disease (OR = 3.67 × 10^7^; 95% CI: 2.67 × 10^7^–5.04 × 10^7^; *p* < 0.001). Similarly, a one-SD decrease in serum albumin (SD = 0.34 g/dL) corresponded to higher odds of periodontal disease (OR = 4.13; 95% CI: 4.11–4.15; *p* < 0.001). For MCHC (SD = 1.02 g/dL), a one-SD increase was also positively associated with periodontal disease (OR = 4.32 × 10^16^; 95% CI: 3.56 × 10^16^–5.24 × 10^16^; *p* < 0.001). Taken together, these SD-scaled findings align with the direction of associations observed in the primary adjusted model and further reinforce the strong gradient between systemic hematologic alterations and periodontal disease.

## 4. Discussion

The biomarker patterns observed in this study are consistent with the broader understanding that periodontitis may produce measurable systemic effects. Elevated WBC levels support the well-established concept that periodontitis contributes to persistent low-grade immune activation. Conversely, reduced serum albumin levels align with their role as a negative acute-phase reactant, suggesting that periodontitis may influence hepatic protein synthesis or increase albumin consumption during inflammatory processes.

Our finding of higher MCHC may indicate alternative erythrocyte responses, possibly linked to oxidative stress, altered red blood cell hydration, or population-level differences captured by NHANES.

Population-based research has consistently reported associations between periodontitis and blood biomarkers. Prior NHANES analyses and international cohort studies have consistently demonstrated that periodontitis contributes to increased circulating WBC as part of an amplified systemic inflammatory response [30,31].

Similarly, the inverse association between serum albumin and periodontitis observed in our analysis is consistent with current evidence. Serum albumin is a well-recognized negative acute-phase reactant, and multiple investigations have reported reduced albumin concentrations among individuals with greater periodontitis or poorer oral health [32,33].

In contrast, our results for the mean corpuscular hemoglobin concentration (MCHC) diverge from most previous studies, which have generally reported lower MCHC values in chronic inflammatory or anemia-of-inflammation states [34,35]. While anemia-related erythropoietic changes are frequently described in periodontitis [36], the elevated MCHC levels identified in our sample represent a departure from this common pattern. This discrepancy may reflect population-specific variability, residual confounding related to unmeasured dietary or hematologic factors, or differences in erythrocyte morphology across demographic subgroups. Given that evidence linking MCHC and periodontitis is extremely limited, our finding contributes novel insight but warrants further investigation in longitudinal and mechanistic studies.

Several mechanistic pathways may explain the association between blood-based biomarkers and periodontitis. Research has shown that PD is initiated by a dysbiotic oral microbiota, particularly Gram-negative anaerobes such as *Porphyromonas gingivalis*, *Treponema denticola*, and *Tannerella forsythia* [37]. These pathogens may stimulate pattern recognition receptors (PRRs), including Toll-like receptors (TLRs), on resident immune and epithelial cells such as neutrophils, monocytes, and lymphocytes [38,39]. Activation of these receptors may trigger the release of pro-inflammatory cytokines (C-reactive protein, IL-6, IL-1β, TNF-α), which may drive chemotactic signaling and attract neutrophils to the periodontal sulcus [38,39].

The bone marrow may also respond with increased production and release of segmented neutrophils, elevating their percentage in peripheral blood and numbers in the gingival crevicular fluid (GCF) [40]. Studies have shown that upon arrival at the periodontal site, neutrophils may attempt to phagocytose periodontal pathogens, initiate the oxidative burst, and produce reactive oxygen species (ROS) [41]. Furthermore, in healthy periodontium, once the microbial threat is cleared, neutrophils may undergo apoptosis and are removed by macrophages, leading to resolution of inflammation [42]. However, in periodontitis, neutrophils may exhibit delayed apoptosis or hyper-responsiveness, resulting in prolonged activation at the site [43]. This chronic activation may result in sustained release of ROS and proteolytic enzymes, contributing to tissue destruction, pocket formation, and bone loss [44]. Additionally, elevated neutrophil counts in peripheral blood may represent a systemic spillover of local periodontitis and a pro-inflammatory systemic phenotype [45,46]. This is particularly relevant for individuals with comorbidities such as diabetes or cardiovascular disease [45,46].

Studies have shown that during periodontitis, monocytes migrating into gingival tissues may differentiate into pro-inflammatory macrophages (M1 type) and osteoclast precursors [47,48,49]. On one hand, M1 macrophages may secrete high levels of IL-1β, TNF-α, and matrix metalloproteinases (MMPs), which may degrade extracellular matrix, connective tissue, and exacerbate alveolar bone loss [47,48,49]. On the other hand, monocytes may also differentiate into osteoclasts in the presence of RANKL (Receptor Activator of Nuclear Factor κB Ligand), driving bone resorption in periodontitis [47,48,49]. This mechanism is crucial for the irreversible alveolar bone loss that characterizes stage III and IV in chronic periodontitis [47,48,49].

Research has demonstrated that in chronic periodontitis, some activated monocytes may spill into systemic circulation, contributing to low-grade systemic inflammation [50,51]. These circulating monocytes may also express high levels of adhesion molecules and infiltrate vascular tissues, and link periodontitis to comorbidities such as cardiovascular disease and diabetes mellitus [50,51]. Recent evidence further suggests that periodontal pathogens may epigenetically reprogram monocytes, promote “trained immunity,” and generate hyper-responsive monocytes upon secondary stimulation [52,53].

Research has shown that pro-inflammatory cytokines such as IL-6, TNF-α, and IL-1β, released from inflamed periodontal tissues, may downregulate hepatic synthesis of albumin [54,55]. This may result in lower serum albumin concentrations in patients with periodontitis compared to periodontally healthy individuals [54,55].

Studies have demonstrated that hypoalbuminemia may reflect malnutrition or frailty, which may compromise oral health and exacerbate periodontal disease [56,57]. Conversely, chronic periodontitis may impair chewing efficiency and nutrient intake, aggravate nutritional deficits and reduce serum albumin levels [58]. As such, albumin may act as a bi-directional marker, linking poor nutrition and periodontitis [58].

Recent evidence has shown that albumin has a major role as a plasma antioxidant, binding free radicals, heavy metals, and advanced glycation end-products (AGEs) [59]. In periodontitis, oxidative stress may be elevated due to overactivation of neutrophils and chronic infection [60]. As a result, consumption of albumin as an antioxidant defense mechanism may deplete its levels in circulation [60]. Recent evidence highlights that antioxidant therapies may help in inflammation resolution [61]. Additionally, studies have shown that low serum albumin is associated with vascular dysfunction, atherosclerosis, and chronic kidney disease, which may share inflammatory pathways with periodontitis [62]. Moreover, periodontitis may amplify systemic endothelial injury, indirectly lowering albumin via the acute-phase response [63]. This may contribute to the oral–systemic connection, where albumin decline mirrors broader inflammatory burden [62,63]. 

**Figure 2:**
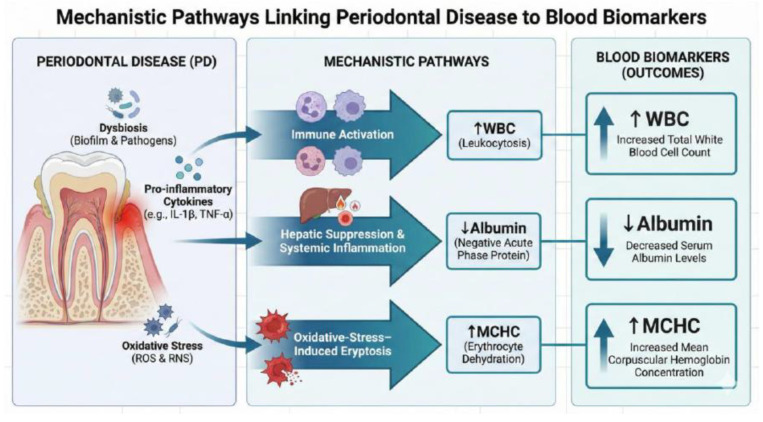
Proposed mechanistic pathways linking periodontitis to systemic hematologic and biochemical alterations. Note: The causal relationship cannot be inferred from this cross-sectional figure.

According to the current literature, one potential explanation for elevated MCHC involves oxidative-stress-mediated erythrocyte dehydration [64,65,66]. Several meta-analyses have demonstrated that chronic periodontitis has been shown to elevate systemic oxidative stress and increase myeloperoxidase activity in saliva and gingival crevicular fluid [64,65,66]. Both processes may contribute to structural injury of erythrocyte membranes [64,65,66]. Mechanistic reviews in hematology indicate that reactive oxygen species may activate cation channels and trigger eryptosis, a form of programmed red blood cell death characterized by cell shrinkage and dehydration [67,68,69]. As erythrocytes lose water and shrink, hemoglobin becomes more concentrated within the cell, even without increasing hemoglobin mass. This process may result in normal or slightly elevated MCHC values [67,68,69]. These data collectively support the biological plausibility that periodontitis-related oxidative stress may contribute to erythrocyte structural injury and eryptosis, potentially altering red blood cell indices. Given the scarcity of MCHC–periodontitis research and inconsistent prior evidence, this association should be interpreted cautiously until replicated in longitudinal or hematologic studies. These mechanistic pathways are summarized in Figure 2.

### 4.1. Limitation of Research

Several limitations of this study should be acknowledged. First, the cross-sectional design of NHANES precludes the determination of causality; thus, the temporal sequence of the association between periodontal disease and systemic biomarker alterations remains uncertain. Second, despite rigorous multivariable adjustment, residual confounding from unmeasured factors, such as detailed dietary micronutrient intake, systemic inflammation, or specific medication use, cannot be excluded. Third, while the use of complete-case analysis carries a risk of selection bias, missingness was minimal (<5%), rendering substantial bias unlikely.

Finally, a specific distinction regarding the MCHC findings warrants consideration. In contrast to prior clinical and case–control studies reporting lower MCHC in periodontitis, this nationally representative analysis identified a positive association. This divergence may reflect differences in population characteristics, such as the generally adequate nutritional status of the NHANES cohort, or potential physiological influences like erythrocyte dehydration and plasma volume contraction.

### 4.2. Future Perspectives

The findings of this study underscore the potential of routinely measured hematological and biochemical parameters—such as white blood cell count, serum albumin, and mean corpuscular hemoglobin concentration (MCHC)—as accessible indicators of systemic alterations associated with periodontal disease. Future research should build upon these observations by employing longitudinal or cohort designs to clarify the temporal direction and causal nature of these associations. Establishing whether systemic inflammatory and hematologic changes precede, coincide with, or follow the onset of periodontal disease will be essential to determine their diagnostic and prognostic utility.

Furthermore, integrative analyses incorporating inflammatory cytokines, oxidative stress biomarkers, and nutritional indicators could provide a more comprehensive understanding of the biological pathways linking oral and systemic health. Multi-omics approaches, including proteomics, metabolomics, and transcriptomics, may also help identify molecular signatures that mediate the interactions between systemic inflammation, erythropoiesis, and periodontal tissue destruction.

Given the cross-sectional nature of the present study, future investigations should also address potential residual confounding from unmeasured lifestyle, dietary, or metabolic variables. In addition, extending this line of inquiry to diverse and underrepresented populations will improve the generalizability of findings and reveal potential population-specific hematologic or inflammatory responses to periodontal pathology.

Lastly, clinical trials evaluating periodontal therapy and its impact on systemic biomarkers could help determine whether improvements in periodontal health translate into measurable hematologic and inflammatory benefits. Such evidence would strengthen the concept of periodontitis as a systemic condition and highlight the role of oral health management in overall disease prevention.

## 5. Conclusions

In summary, this cross-sectional analysis of nationally representative NHANES 2013–2014 data revealed positive associations between periodontal disease and systemic blood biomarkers. Individuals with periodontitis exhibited higher white blood cell counts and lower serum albumin levels, consistent with a heightened systemic inflammatory state. Interestingly, mean corpuscular hemoglobin concentration (MCHC) showed a positive association with periodontal disease, diverging from findings of smaller clinical studies that have typically reported reduced MCHC in chronic inflammation. These results reinforce the concept that periodontal disease extends beyond local tissue destruction and is linked to measurable systemic hematologic and biochemical alterations. These findings underscore the potential clinical utility of routine blood biomarkers as accessible indicators of systemic inflammation associated with periodontal disease. Nonetheless, given the cross-sectional nature of the data, causal relationships cannot be inferred. Future longitudinal and interventional studies are warranted to clarify temporal dynamics, explore mechanistic pathways, and determine whether periodontal treatment can modulate these systemic biomarkers, ultimately improving both oral and general health outcomes.

## Figures and Tables

**Table 1:** Survey-weighted sample characteristics of U.S. adults aged ≥ 30 years by periodontal disease status, NHANES 2013–2014.

Covariate	No Periodontitis (%) (n = 2553)	Periodontitis (%) (n = 2116)	*p*-Value
Sex			<0.001
male	43.1%	57.2%	
female	56.9%	42.8%	
Age			<0.001
30–34	12.0%	8.8%	
35–49	34.6%	30.8%	
45–64	30.7%	36.6%	
65+	22.7%	23.7%	
Race			<0.001
Non-Hispanic White	71.6%	60.2%	
Non-Hispanic Black	9.6%	14.0%	
Hispanic	11.6%	17.1%	
Non-Hispanic Asian	5.0%	6.1%	
Other	2.2%	2.6%	
Education			<0.001
<High School	12.9%	20.7%	
High School/GED	18.9%	26.9%	
College or More	68.2%	52.4%	
Smoking			<0.001
No	58.6%	45.8%	
Yes	41.4%	54.2%	
Family Income Ratio to FPL			<0.001
<138%	18.6%	28.9%	
138–399%	34.8%	40.8%	
>400%	46.5%	30.3%	
Any disease			0.149
No	55.5%	52.9%	
Yes	44.5%	47.1%	

Footnotes. All estimates are survey-weighted using NHANES Mobile Examination Center (MEC) examination weights (WTMEC2YR) and account for the complex multistage probability sampling design, including strata and primary sampling units. Percentages represent weighted proportions of the U.S. adult population; unweighted sample sizes (n) are shown in column headers. *p*-values for categorical variables are derived from the Rao–Scott F-adjusted chi-square test; *p*-values for continuous variables (biomarkers) are derived from survey-weighted linear regression models. “Any disease” includes self-reported physician diagnoses of diabetes, myocardial infarction, coronary heart disease, congestive heart failure, stroke, chronic kidney disease, liver disease, or cancer. Periodontal disease was classified according to the CDC/AAP surveillance case definition and dichotomized into “with” and “without” periodontitis.

**Table 2:** Survey-weighted mean levels of blood biomarkers (WBC, serum albumin, and MCHC) among U.S. adults aged ≥30 years by periodontal disease status, NHANES 2013–2014.

Biomarker	No Periodontitis (Mean ± SD) (n = 2553)	Periodontitis (Mean ± SD) (n = 2116)	*p*-Value
WBC Count (10^3^ cells/µL)	7.21 ± 0.07	7.57 ± 0.08	0.002
Serum Albumin (g/dL)	4.26 ± 0.01	4.22 ± 0.01	0.002
MCHC (g/dL)	33.80 ± 0.08	33.98 ± 0.11	0.052

Footnotes: Values represent survey-weighted means ± standard errors (SE), estimated using NHANES MEC examination weights (WTMEC2YR). *p*-values are derived from survey-weighted linear regression models testing mean differences in biomarker levels between periodontal groups. Biomarker distributions were evaluated for linearity using restricted cubic splines; subsequent regression models incorporated standardized units where appropriate.

**Table 3:** Stepwise survey-weighted multiple logistic regression models showing associations between blood biomarkers and periodontal disease.

		Composite		
Covariate	Odds Ratio	Confidence Interval		*p* Value
		Lower	Upper	
White blood cells	1.075	1.044	1.107	<0.001
Serum albumin	0.760	0.615	0.939	0.011
MCHC	1.138	1.062	1.219	<0.001
Sex (reference: Male)	0.551	0.481	0.631	<0.001
Age (reference: 30–34)				
35–49	1.256	0.993	1.588	0.056
45–64	1.953	1.532	2.489	<0.001
>65	1.742	1.346	2.255	<0.001
Education (reference: <high school)				
High school/GED	1.006	0.832	1.217	0.947
Some college or more	0.734	0.616	0.874	0.001
Poverty (reference: <138 FPL)				
138–399%	0.883	0.754	1.035	0.127
>400%	0.608	0.511	0.724	<0.001
Race (reference: Non-Hispanic White)				
Non-Hispanic Black	1.927	1.600	2.321	<0.001
Hispanic	1.689	1.416	2.014	<0.001
Non-Hispanic Asian	1.811	1.455	2.253	<0.001
Other	1.338	0.909	2.118	0.128
Smoking (reference: No Smoking)	1.311	1.146	1.500	<0.001
Any Disease (reference: No Disease)	0.906	0.781	1.050	0.190

## Data Availability

The data used in this article are publicly available and can be found at the Centers for Disease Control and Prevention (CDC) National Center for Health Statistics: National Health and Nutrition Examination Survey (NHANES) Questionnaires, Datasets, and Related Documentation, available at the following website: https://wwwn.cdc.gov/nchs/nhanes/ (accessed on 21 September 2025).

## Supplementary Materials

The following supporting information can be downloaded at https://www.mdpi.com/article/10.3390/biomedicines13122991/s1.

## Abbreviations

The following abbreviations are used in this manuscript:

PD	Periodontal disease
WBC	White blood cell
MCHC	Mean corpuscular hemoglobin concentration
ROS	Reactive oxygen species
CDC/AAP	Centers for Disease Control and Prevention/American Academy of Periodontology
