# Supplementary material for: Association Between Periodontal Disease and Blood Biomarkers in U.S. Adults: A Cross-Sectional Study
Source: Biomedicines. 2025 Dec 5;13(12):2991. doi: 10.3390/biomedicines13122991 (PMC12731155; doi:10.3390/biomedicines13122991)
Supplement: Supplementary file 1 [file biomedicines-13-02991-s001.zip › biomedicines-3961791-supplementary.pdf]

Table S1. Number of non-missing observations and percentage of missing values for analytic variables (NHANES 2013–2014, n = 4669).

Variable	Non-missing N	Missing N	% Missing
**Periodontal disease status**	4669	0.00	0.0%
**WBC count (103 cells/ML)**	4504	165	3.5%
**Serum albumin (g/dL)**	4449	220	4.7%
**MCHC (g/dL)**	4504	165	3.5%
**Age (years)**	4669	0.00	0.0%
**Sex**	4669	0.00	0.0%
**Race**	4669	0.00	0.0%
**Education level**	4665	4	0.1%
**Income to poverty ratio**	4669	0.00	0.0%
**Smoking status**	4669	0.00	0.0%
**Any disease (comorbidity)**	4655	14	0.3%

Table S2. Survey-Weighted Logistic Regression Estimates for Biomarker Effects Expressed per Standard Deviation (SD) Increase.

Biomarker	Standard Deviation	OR per SD Increase	95% CI	P-Value
**WBC Count (10^3 cells/μL)**	2.2898	3.67×10^7	$2.67 \times 10^7 – 5.04 \times 10^7$	<0.001
**Serum Albumin (g/dL)**	0.3403	4.13	4.10 – 4.15	<0.001
**MCHC (g/dL)**	1.0227	4.32×10^{16}	$3.56 \times 10^{16} – 5.24 \times 10^{16}$	<0.001

Footnotes:

1. Values derived from survey-weighted linear combination estimates (lincom) using SD-scaled biomarker coefficients.
2. SD values computed using estat sd following survey-weighted mean estimation.
3. Extremely large ORs for WBC and MCHC reflect the magnitude of the SD-scaled coefficients and do not indicate model instability.
4. These results are directionally consistent with the main regression model, confirming robustness of associations.

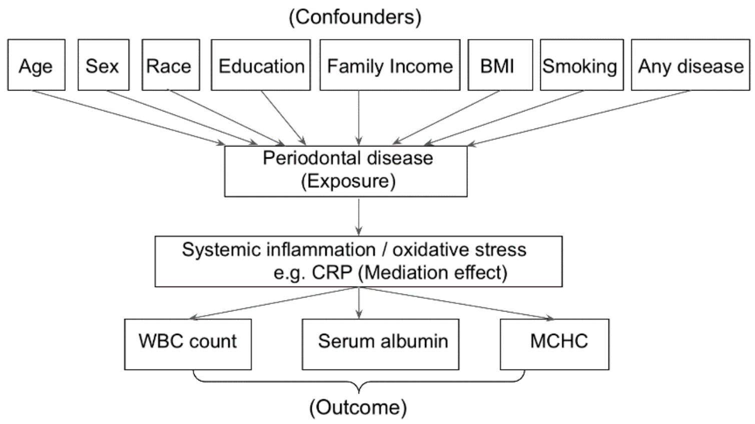

Figure S1. Directed Acyclic Graph (DAG) illustrating the mediation effect of systemic inflammation/oxidative stress in the relationship between periodontal disease and blood biomarkers.
